# Participation of 5-lipoxygenase and LTB4 in liver regeneration after partial hepatectomy

**DOI:** 10.1038/s41598-019-54652-7

**Published:** 2019-12-03

**Authors:** Florencia Lorenzetti, Marina Cecilia Vera, María Paula Ceballos, María Teresa Ronco, Gerardo Bruno Pisani, Juan Alberto Monti, Alvaro Lucci, Carla Gabriela Comanzo, Thierry Tordjmann, María Cristina Carrillo, Ariel Darío Quiroga, María de Luján Alvarez

**Affiliations:** 1 Instituto de Fisiología Experimental (IFISE), Facultad de Ciencias Bioquímicas y Farmacéuticas, CONICET, UNR, Suipacha 570 (S2002LRL), Rosario, Argentina; 2 Área Morfología, Facultad de Ciencias Bioquímicas y Farmacéuticas, UNR, Suipacha 570 (S2002LRL), Rosario, Argentina; 3 INSERM U1174, Université Paris Saclay, bât. 443, 91405 Orsay, France; 4 Centro de Altos Estudios en Ciencias Humanas y de la Salud (CAECIHS) Sede Regional Rosario, Universidad Abierta Interamericana, Av. Pellegrini 1618 (S2000BUG), Rosario, Argentina

**Keywords:** Liver, Physiology

## Abstract

Regeneration is the unmatched liver ability for recovering its functional mass after tissue lost. Leukotrienes (LT) are a family of eicosanoids with the capacity of signaling to promote proliferation. We analyzed the impact of blocking LT synthesis during liver regeneration after partial hepatectomy (PH). Male Wistar rats were subjected to two-third PH and treated with zileuton, a specific inhibitor of 5-lipoxygenase (5-LOX). Our first find was a significant increment of intrahepatic LTB4 during the first hour after PH together with an increase in 5-LOX expression. Zileuton reduced hepatic LTB4 levels at the moment of hepatectomy and also inhibited the increase in hepatic LTB4. This inhibition produced a delay in liver proliferation as seen by decreased PCNA and cyclin D1 nuclear expression 24 h post-PH. Results also showed that hepatic LTB4 diminution by zileuton was associated with a decrease in NF-ĸB activity. Additionally, decreased hepatic LTB4 levels by zileuton affected the recruitment of neutrophils and macrophages. Non-parenchymal cells (NPCs) from zileuton-treated PH-rats displayed higher apoptosis than NPCs from PH control rats. In conclusion, the present work provides evidences that 5-LOX activation and its product LTB4 are involved in the initial signaling events for liver regeneration after PH and the pharmacological inhibition of this enzyme can delay the initial time course of the phenomenon.

## Introduction

The liver has the exceptional ability to regenerate after surgical removal or a toxic insult. Liver regeneration following partial hepatectomy (PH) is an extremely complex and fine-orchestated process. Many signaling cascades are associated with this phenomenon, including cytokines, growth factors, microRNAs, extracellular matrix remodeling signals, which are turned on and off at particular times. After a resection, a complex process begins to restore lost liver mass; at the same time and during the regenerative process, the organ provides full assistance to body homeostasis^1–3^. Clinically, the procedure is performed in humans for resecting solitary liver tumors, repairing injury, as well as for living-donor liver transplantation. The most widely used model in experimental hepatology for studying regenerative liver growth is two-thirds PH in rodents, in which liver initial functional mass is restored in few days^4^.

Eicosanoids are lipid mediators generated from arachidonic acid, which is released by phospholipase A2 enzyme from membrane phospholipids. The three major pathways for eicosanoids synthesis are lipoxygenase (LOX), cyclooxygenase (COX), and epoxygenase. Leukotrienes (LT) are synthesized via 5-lipoxygenase (5-LOX) pathway, which generates biologically inactive leukotriene A4 (LTA4)^5^. LTA4 hydrolase (LTA4-H) catalyzes the hydrolysis of LTA4 to produce leukotriene B4 (LTB4). A second LTA4 metabolic pathway is the production of leukotriene C4 (LTC4) by the enzyme LTC4 synthase (LTC4-S)^6^. Despite LT were firstly described to be produced by leukocytes, it is now known that they are produced by other cell types. In liver, Kupffer cells are the only cell type endowed with a metabolically active 5-LOX pathway^7^.

LTB4 was classically described as a pro-inflammatory mediator, promoting antimicrobial defense and modulating the immune system. When LTB4 is formed in excess or improperly, it can participate in the pathogenesis of chronic inflammatory conditions^8^. In this sense, 5-LOX inhibition is a strategy for stopping LTB4-driven disease progression. Zileuton is a selective and reversible 5-LOX inhibitor. It is the only commercially available 5-LOX inhibitor used for inflammatory airway diseases treatment. After oral administration in humans, zileuton is rapidly absorbed, and its plasma concentration peaks at 1–3 h after dose and correlates with LTB4 inhibition^9,10^.

On the other hand, it is known that LT promote proliferation of tumor cells. An increase in 5-LOX expression and activity was described in certain cancers, while its inhibition was found to cause apoptosis^11^. Interestingly, this proliferative role has been essentially attributed to LTB4^12–15^. Particularly in hepatocellular carcinoma HepG2 cells, LTB4 acts as an activator of NF-kB, a critical factor for cell survival^16^. In liver proliferation, it has been highlighted the association between genes related to lipid metabolism and liver regeneration after PH. Particularly, genes that promote LT synthesis, including *ALOX5*, are up-regulated during the initial times after PH in rat liver^17^. However, the connection between this increase and liver regeneration was not explored. The aim of present study was to evaluate the changes in LT producing-enzymes during liver regeneration and the participation of LT in the phenomenon. Using the specific 5-LOX inhibitor, zileuton, we analyzed the impact of blocking the LT producing pathway during liver proliferation after PH.

## Materials and Methods

### Chemicals

Zileuton was obtained from Hangzhou Starshine Pharmaceutical Co., LTD. Collagenase type IV from Clostridium histolyticum was purchased from Sigma-Aldrich. Antibodies against Cyclin D1, Lamin A/D, 5-LOX, LTA4-H, LTC4-S, CD68, Bax, and Bcl-2 used for western blot studies were from Santa Cruz Biotechnology. Anti- Ikβ-α was purchased from Cell Signalling Technologies, anti-CD11b from Abcam, anti-CLEC 4F from R&D Systems and anti-β-actin was from Sigma.

### Animals and surgery

Adult male Wistar rats weighing 250–280 g were maintained in a room at constant temperature with a 12 h light-dark cycle, with food and water supplied ad libitum. Animal work was performed at the Institute of Experimental Physiology (IFISE, Faculty of Biochemical and Pharmaceutical Sciences, CONICET, National University of Rosario). Experimental protocols were performed according to the National Institutes of Health guide for the care and use of Laboratory animals (NIH Publication 25–28, revised 1996) and approved by the local animal care and use committee: “Bioethical Committee for the Care and Use of Laboratory Animals” from Faculty of Biochemical and Pharmaceutical Sciences of the National University of Rosario (Permission 108/2014, FBioyF, UNR). Animals were anesthetized with ketamine/xylazine (100 and 3 mg/Kg body weight, respectively) and two-thirds PH was performed as previously described by Higgins and Anderson^18^. Also, a simulated surgery was carried out in control animals (sham group, n = 15). Vehicle (saline solution/ethanol, n = 18) or zileuton at the doses of 10 (n = 4) or 40 mg/Kg body weight (n = 18) were administered by gavage two hours before PH in order to obtain the maximal 5-LOX inhibition at the time of surgery. Zileuton dose of 10 mg/Kg was used in rats to treat inflammatory injuries in several organs such as lung and kidney^19,20^; whereas 40 mg/Kg zileuton was comparable to human daily intake for the treatment of airway inflammatory conditions^10^. Animals were anesthetized and euthanized by exsanguination at 1, 5, 24 and 48 h post-PH and blood samples were collected and livers were removed to be processed (n = 6 for animals euthanized at 24 h post-PH, and n = 4 for animals euthanized at 1, 5 and 48 h post-PH).

Liver weight/body weight ratio [(liver weight/body weight) × 100] was calculated and used as a measurement of total volume of regenerating liver at the different times post-PH.

### Enzymes activities determination

Alanine and aspartate aminotransferases (ALT and AST, respectively) and alkaline phosphatase (ALP) were determined spectrophotometrically in fresh serum by commercial kits (Wiener Lab, Rosario, Argentina).

### Histology and immunohistochemical studies

Liver tissue fixed in 10% neutral buffered formalin and embedded in low melting paraffin was used for immunohistochemical detection of proliferating cell nuclear antigen (PCNA), cyclin D1 and myeloperoxidase (MPO). Liver sections were deparaffinized, rehydrated and endogenous peroxidase was blocked. Slides were boiled in sodium citrate buffer pH 6 for antigen retrieval and incubated overnight with the primary antibodies (anti-PCNA, Santa Cruz; anti-cyclin D1, Abcam and anti-MPO, Dako). CytoScan HRP Detection System (Cell Marque) was used for detection of positive labelled cells. The PCNA proliferative index was defined as the number of proliferative cells (in G_1_, S, G_2_ and M phases) per 100 hepatocytes counted in ten high-power fields. Proliferating hepatocytes in each phase of the cell cycle were also determined by a blinded histological analysis based on the specific PCNA staining patterns, as previously described^21,22^. MPO-positive cells were counted in 20 randomly selected fields (200X).

Liver sections were also assessed for histological changes by Hematoxylin and Eosin (H&E) staining at 24 h post-PH. Mitotic figures were evaluated by examination of H&E sections. Number of mitotic figures was counted in 20 representative 200X fields.

For immunofluorescence studies, cryostat liver sections were fixed with ice-cold 4% paraformaldehyde, permeabilized with 0.2% Triton X100 in PBS, blocked and overnight incubated with anti-rat neutrophil FITC-conjugated antibody (LS-C348005, LSBio). Detection of bound antibody was achieved by immunofluorescence in a Nikon C1 Plus microscope. Neutrophils were counted in 20 randomly selected fields.

### ELISA assay for hepatic LTB4 and LTC4 quantification

LTB4 and LTC4 were determined in liver extracts with commercial ELISA kits (NEOGEN Corporation). LT were purified in C18 Sep-Pak® light Columns (Waters® Corporation), eluted with ethyl acetate (LTB4) or methanol (LTC4), evaporated with nitrogen gas and dissolved in the ELISA’s extraction buffer. The ELISAs were performed as indicated in the product datasheet.

### Western blot analysis

Liver tissue lysates and mitochondrial and nuclear extracts were prepared as previously described^21,23^. Proteins were quantified according to Lowry *et al*.^24^ and western blot studies were performed as formerly described^21^.

### RNA isolation, cDNA synthesis and RT-qPCR analysis

RNA was isolated from rat liver tissue by the TriZOL method (Life Technologies Inc.) according to the manufacturer’s instructions. One microgram of total RNA was treated with DNase I (Thermo Fisher Scientific) and cDNA was made using an oligo-dT primer and M-MLV reverse transcriptase (Promega). PCR assay was performed using an Mx3000P Real-Time Thermocycler (Stratagene) with EvaGreen® dye (HOT FIREPol® EvaGreen® qPCR Mix Plus (ROX); Solis BioDyne). Primer sequences are shown in Supplementary Information. PCR reactions were initiated by incubation at 95 °C for 12 min, followed by 40 cycles at 95 °C for 15 s, 60 °C for 30 s and 72 °C for 30 s. Relative changes in gene expression were determined by using the 2^−ΔΔCt^ method^25^.

### NF-kB activity assay

The NF-kB activity was determined in nuclear extracts by a colorimetric reaction using NFκB p50/p65 EZ-TFA Transcription Factor Assay (Millipore Corporation), following the instructions manual of the kit.

### Liver leukocytes isolation

Liver leukocytes were isolated as previously described, with slight modifications^26^. Briefly, 5 h after hepatectomy, livers from PH (n = 4) and PHZi rats (n = 4) were pressed through a 100 µm cell strainer (Jet Bio-Fil). Liver cells were suspended in Hank’s Balanced Salt Solution (HBSS) and centrifuged at 500 *g* for 5 min. Pellets were re-suspended in 35% Percoll (Sigma) containing 100 U/mL heparin and centrifuged at 800 *g* for 20 min at room temperature. The cell pellets containing leukocytes were collected and re-suspended in red blood cell lysis solution. After 5 min incubation on ice, cells were washed twice in HBSS. For analysis of neutrophil markers MPO and elastase, 10^6^ cells were subjected to RNA isolation and RT-qPCR, as previously described.

### Isolation of non-parenchymal cells and hepatocytes

Non-parenchymal cells (NPCs) and hepatocytes were isolated from sham (n = 3) and PH and PHZi rats 1, 5, and 24 h (n = 4 per group at each time) after surgery as previously described, with few modifications^27^. Briefly, animals were anesthetized with ketamine/xylazine and after catheterization of the portal vein, the liver was perfused with collagenase type IV. NPCs were separated from hepatocytes and counted, and cell viability was analyzed by trypan blue exclusion test. Purity of NPCs fraction from hepatocytes markers was checked by western blot (data not shown).

### Caspase-3 activity

Caspase-3 activity was determined in lysates of hepatocytes and NPCs using Enz Chek Caspase-3 Assay kit #1 (Molecular Probes Inc, Eugene, OR, USA), following the manufacturer’s suggestions.

### Annexin V/propidium iodide assay

NPCs (1,5 × 10^6^ cells) were used to assess the apoptotic cell death by AnnexinV/propidium iodide staining (FITC Annexin V Apoptosis Detection Kit II; BD Biosciences) coupled to flow cytometry analysis (BD FACSAria™ II cell sorter flow cytometer, BD Biosciences), as previously described^28^.

### Statistical analysis

Results are expressed as mean ± SEM. Statistical significance was evaluated by unpaired two- tailed Student’s test or by one-way ANOVA followed by Tukey test. Differences were considered significant when p < 0.05.

## Results

### Hepatic enzymes activities did no change after oral administration of zileuton

The activities of ALT, AST and ALP increased after PH and peaked 24 h post-PH. On the other hand, there was no statistical difference between the groups treated with vehicle or zileuton, indicating that zileuton dosage did not affect serum markers of liver function (Table 1). Indeed, zileuton-treated sham animals showed no difference in the activities of these liver markers as in any other parameter measured further on, thus, this group will not be included in the description of the results.

**Table 1:** AST, ALT and ALP enzyme activities.

Experimental group		AST (U/L)	ALT (U/L)	ALP (U/L)
Sham		232,5 ± 24,0	162,2 ± 36,8	260,8 ± 52,3
1 h post-PH	PH	201,7 ± 38,5	146,0 ± 27,0	223,0 ± 24,1
	PHZi	174,0 ± 50,0	131,2 ± 33,9	258,3 ± 30,4
5 h post-PH	PH	784,0 ± 159,7*	1376,7 ± 269,8*	427,1 ± 38,8*
	PHZi	966,0 ± 175,1*	1886,9 ± 139,9*	310,6 ± 69,5*
24 h post-PH	PH	1211,0 ± 300,3*	2512,9 ± 197,3*	375,7 ± 20,7*
	PHZi	1213,7 ± 200,5*	1946,0 ± 23,0*	398,5 ± 67,4*
48 h post-PH	PH	410,8 ± 101,8*	518,9 ± 208,8*	524,1 ± 33,6*
	PHZi	673,0 ± 84,2*	703,7 ± 138,0*	484,3 ± 55,2*

AST, ALT and ALP enzyme activities in plasma samples of sham and partial-hepatectomized animals treated with vehicle (PH) or zileuton 40 mg/Kg body weight (PHZi). Data were expressed in units per litre of sample (U/L) and represent media ± SEM (*n* ≥ 4). *p < 0.05 vs. Sham.

### Zileuton reduced liver proliferation after PH

**Figure 1:**
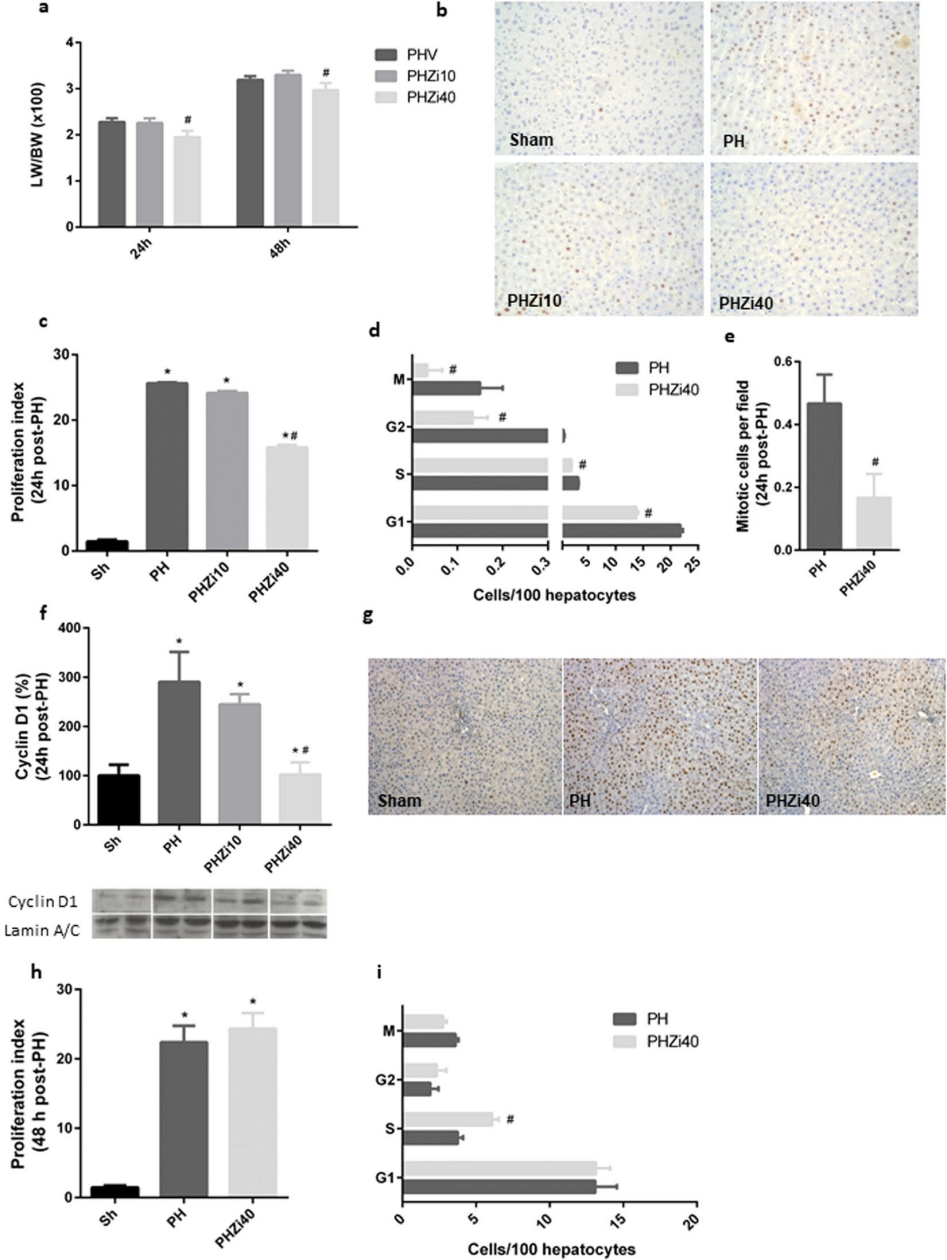
Effect of oral administration of zileuton in liver proliferation in PH-rats. (**a**) Liver weight to body weight (LW/BW) ratio 24 and 48 h post-PH. (**b**) Representative images of Proliferating Cell Nuclear Antigen (PCNA) immunohistochemistry from rat liver tissue 24 h post-PH obtained by optical microscopy (200X). (**c**) Proliferation Index (PI) determined by PCNA staining 24 h post-PH. PI was calculated as the number of cells in G_1_, S, G_2_ and M phases of the cell cycle per 100 hepatocytes. (**d**) Number of PCNA-positive cells in each phase of the cell cycle per 100 hepatocytes 24 h post-PH. (**e**) Quantification of mitotic figures by analysis of H&E images 24 h post-PH. Analysis of cyclin D1 24 h post-PH by (**f**) immunoblotting studies in liver nuclear extracts and (**g**) immunohistochemistry (100X). Selected lanes were cropped from different parts of the same gel and they are shown after cropping, aligning and separating them by white space. Full-length blots are available in Supplementary Information. (**h**) Proliferation Index determination by PCNA immunohistochemistry in 48 h post-PH rat liver tissue. (**i**) PCNA-positive cells in each phase of the cell cycle per 100 hepatocytes 48 h post-PH. Sh: sham animals, PH: partial-hepatectomized animals treated with vehicle, PHZi10: PH-animals treated with zileuton 10 mg/Kg body weight, PHZi40: PH-animals treated with zileuton 40 mg/Kg body weight. Bars represent mean ± SEM (*n* ≥ 4 per experimental group). *p < 0.05 vs. Sh, ^#^p < 0.05 vs. PH.

In order to establish if liver regeneration was affected by zileuton, we first analyzed liver weight to body weight (LW/BW) ratio after PH. LW/BW ratio was significantly reduced in rats treated with 40 mg/Kg zileuton at 24 and 48 h post-PH, showing a decrease of 15 and 10%, respectively (Fig. 1a). Next, we analyzed PCNA-positive hepatocytes by immunohistochemistry 24 h post-PH, time in which rat hepatocytes displayed an initial peak of DNA synthesis^29^. Figure 1b shows representative images of PCNA staining, with a clear decrease in PCNA-positive hepatocytes in PH-rats treated with 40 mg/Kg zileuton. Proliferative index analysis 24 h post-PH showed a significant decrease in PH-animals treated with 40 mg/Kg zileuton, with less hepatocytes in each phase of the cell cycle (Fig. 1c,d). In accordance, the number of mitotic figures was reduced in PH-rats treated with zileuton (Fig. 1e). As expected, and supporting these findings, immunoblotting and immunohistochemistry for cyclin D1 24 h post-PH showed that nuclear levels of cyclin D1 increased in the PH-rats, but were notably reduced in zileuton-treated PH-rats (Fig. 1f,g). As 10 mg/Kg zileuton had effect neither on LW/BW ratio nor on liver proliferation 24 h after PH, we continued using 40 mg/Kg zileuton throughout the study.

On the other hand, there was no difference in the proliferation index between zileuton and vehicle-treated PH groups 48 h post-PH (Fig. 1h), indicating a partial recovery of regeneration. Interestingly, there was an accumulation of cells in the S phase (Fig. 1i), which could be a consequence of the delay in proliferation. In accordance, there was no significant difference in LW/BW ratio 168 h after surgery (data not shown), indicating that zileuton affects only the initial stage of regeneration, but final liver weight recovery can be reached.

Clearly, a single dose of 40 mg/Kg zileuton 2 h before PH delays the initial time course of liver regeneration, significantly affecting proliferation 24 h post-surgery, the time in which hepatocytes DNA synthesis peaks^1^. At later times, the delay in liver proliferation is rescued. Despite additional doses would be necessary to determine if a sustained zileuton treatment impacts on final liver recovery, the results presented in this work using zileuton for inhibition of 5-LOX at the onset of liver regeneration shows that 5-LOX pathway can take part in the regenerative process.

Additionally, the specificity of the role of LTB4 in liver regeneration after PH was established using an inhibitor of LTA4-H. PCNA-positive hepatocytes 24 h post-PH significantly decreased in animals treated with LTA4-H inhibitor, confirming that the specific inhibition of hepatic LTB4 affected liver regeneration after PH (Supplementary Fig. 1).

### Hepatic LTB4 levels augmented 1 h after PH and zileuton treatment affected this increase

**Figure 2:**
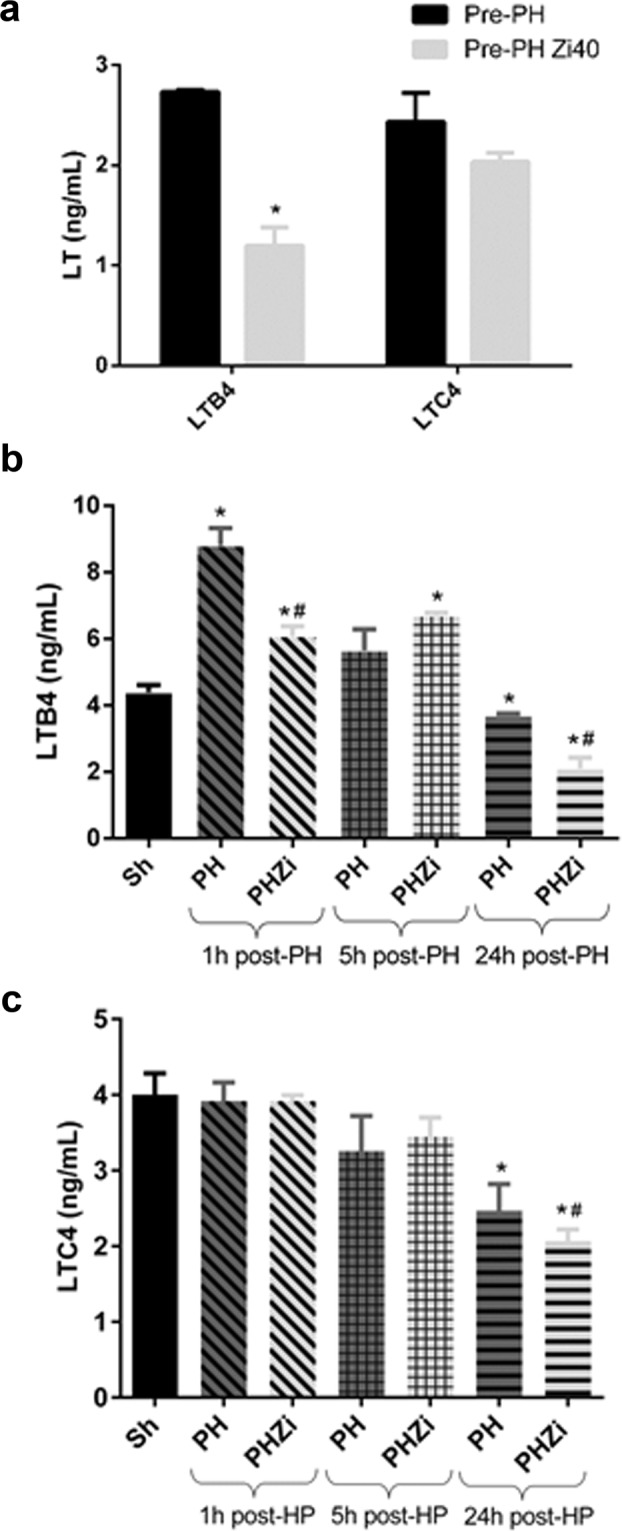
Hepatic LTB4 and LTC4 levels detected by ELISA. (**a**) LTB4 and LTC4 levels in the removed lobes (time zero for surgery and 2 h after zileuton administration). (**b**) LTB4 and (**c**) LTC4 hepatic levels at 1, 5 and 24 h post-partial hepatectomy (PH) in animals treated with vehicle or zileuton 40 mg/Kg body weight. Pre-PH: Removed tissue from animals treated with vehicle, Pre-PHZi40: Removed tissue from animals treated with zileuton 40 mg/Kg body weight, Sh: sham animals, PH: partial-hepatectomized animals treated with vehicle, PHZi: PH-animals treated with zileuton 40 mg/Kg body weight. Bars represent mean ± SEM (*n* = 4 per experimental group). *p < 0.05 vs. Sh or Pre-PH, ^#^p < 0.05 vs. PH for each time.

To determine the effectiveness of zileuton in the inhibition of hepatic 5-LOX activity, we measured the levels of the main LT (LTB4 and LTC4) in the removed liver lobules (maximum zileuton serum concentration and time 0 h for hepatectomy). Figure 2a shows that animals treated with zileuton exhibited more than 50% reduction in hepatic LTB4 levels, which means that at the time of surgery LTB4 levels were below control levels, with no significant changes in LTC4 levels.

Surprisingly, hepatic LTB4 levels showed a two-fold increase 1 h post-PH compared to the sham group. Five hours post-PH LTB4 levels showed a non-significant increase, and 24 h post-PH LTB4 levels were significantly reduced, as seen in Fig. 2b. Zileuton administration partially blocked the increase in hepatic LTB4 1 h post-PH. By contrast, the amount of hepatic LTC4 did not change at early times, but its levels were reduced 24 h post-PH (Fig. 2c).

### 5-LOX along with other enzymes of LT synthesis changed their expression during liver regeneration

**Figure 3:**
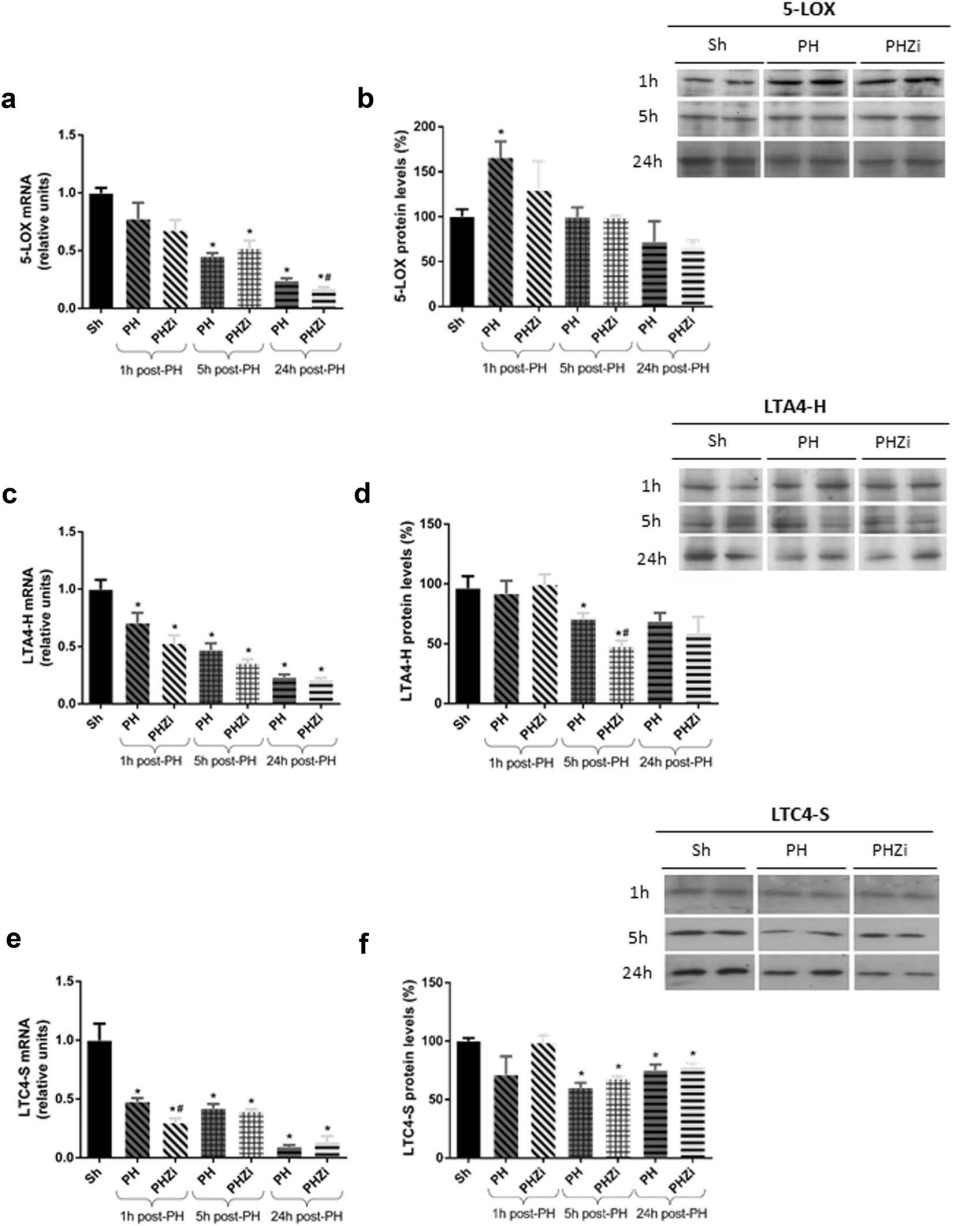
mRNA and protein expression of main enzymes for LT synthesis in rat liver 1, 5 and 24 h post-PH in animals treated with vehicle or zileuton. RT-qPCR of (**a**) 5-LOX, (**c**) LTA4-H and (**e**) LTC4-S. Immunoblotting of (**b**) 5-LOX, (**d**) LTA4-H and (**f**) LTC4-S. Selected lanes were cropped from different gels and they are shown after cropping, aligning and separating them by white space. Full-length blots are available in Supplementary Information. The amount of every protein band was corrected by β-actin but not shown in order to avoid a more complex image. Sh: sham animals, PH: partial-hepatectomized animals treated with vehicle, PHZi: PH-animals treated with zileuton 40 mg/Kg body weight. Bars represent mean ± SEM (*n* = 4 per experimental group). *p < 0.05 vs. Sh, ^#^p < 0.05 vs. PH for each time.

Consistent with hepatic levels of LTB4 1 h post-PH, we observed an increase in 5-LOX protein expression at that time, with no changes in mRNA levels (Fig. 3a,b). The difference between the expression of mRNA and protein may be due to the fact that increased protein expression could be regulated by a post-transcriptional mechanism or because 5-LOX mRNA could increase at shorter times than 1 hour post-PH. At longer times, protein levels of 5-LOX returned to those observed in sham animals, with a trend to be reduced 24 h post-PH. Besides, 5-LOX mRNA expression was significantly reduced 5 and 24 h post-PH. Remarkably, 5-LOX induction during the initial times of liver regeneration after PH seems to be a general event in rodents since we observed an increase in 5-LOX protein and mRNA levels also in mice 2 h after PH, becoming lower at later times (Supplementary Fig. 2a,b).

LTA4-H mRNA expression was reduced after PH at all the studied times. Protein levels of this enzyme tended to be diminished, although they were only significantly different 5 h post-PH (Fig. 3c,d). Likewise, LTC4-S expression remained low throughout the regeneration study (Fig. 3e,f). These patterns were similar to those observed in mice (Supplementary Fig. 2c–f).

### 5-LOX inhibition by zileuton reduced the activation of the transcription factor NF-kB

**Figure 4:**
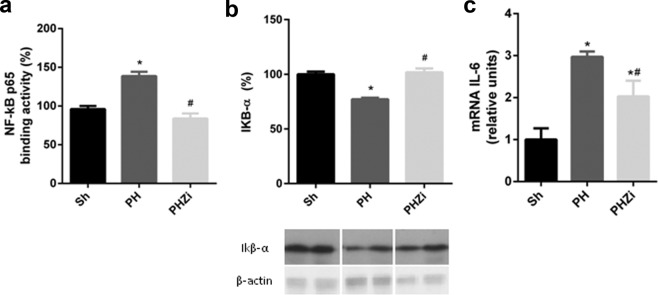
Analysis of zileuton effect on NF-κB activation 1 h post-PH. (**a**) NF-κB binding activity measured with a commercial kit. (**b**) Immunoblotting of the NF-κB inhibitor, IκB-α, in total lysates. (**c**) Analysis of mRNA expression by RT-qPCR of IL-6. Selected lanes were cropped from different parts of the same gel and they are shown after cropping, aligning and separating them by white space. Full-length blots are available in Supplementary Information. Sh: sham animals, PH: partial-hepatectomized animals treated with vehicle, PHZi: PH-animals treated with zileuton 40 mg/Kg body weight. Bars represent mean ± SEM (*n* = 4 per experimental group). *p < 0.05 vs. Sh, ^#^p < 0.05 vs. PH.

During the first hours after PH (priming phase), NF-kB transcription factor is activated in hepatocytes and NPCs. Upon activation, NF-kB induces the production of interleukin-6 (IL-6), a cytokine that mediates hepatocytes proliferation^4^. Thus, we sought to evaluate whether 5-LOX inhibition by zileuton affected or not NF-kB pathway. As expected, the activity of this protein in liver nuclear extracts was increased 1 h post-PH, but it was significantly reduced in liver nuclear extracts from rats treated with zileuton (Fig. 4a). Also, levels of the inhibitor of NF-kB, Ikβ-α, were significantly increased in the animals treated with zileuton (Fig. 4b).

Additionally, we determined IL-6 mRNA levels in NPCs 1 hour after PH. Results are shown in Fig. 4c. IL-6 mRNA levels significantly increased after PH and this increment was partially blocked by zileuton, indicating that IL-6 production by macrophages can be affected by zileuton treatment. This result was in accordance with the decrease in NF-kB activation observed in this experimental group. Additionally, hepatic mRNA levels of TNF-α, IL-1β and IL-10 were analyzed and they did not show differences between PH and PHZi groups (data not shown).

After PH, multiple signaling mechanisms are turned on and off at particular times to ensure optimal liver regeneration^30^. Among these pathways, Wnt/β-catenin was explored since it is turned on when LTB4 levels are increased after PH. Interestingly, inhibition of hepatic LTB4 by zileuton decreased Wnt2 mRNA expression in NPCs and increased hepatic inactive β-catenin levels (Supplementary Fig. 3). These results are indicative that zileuton treatment impacts on Wnt/β-catenin signaling after PH.

### Reduced hepatic LTB4 levels by zileuton affected the recruitment of neutrophils

**Figure 5:**
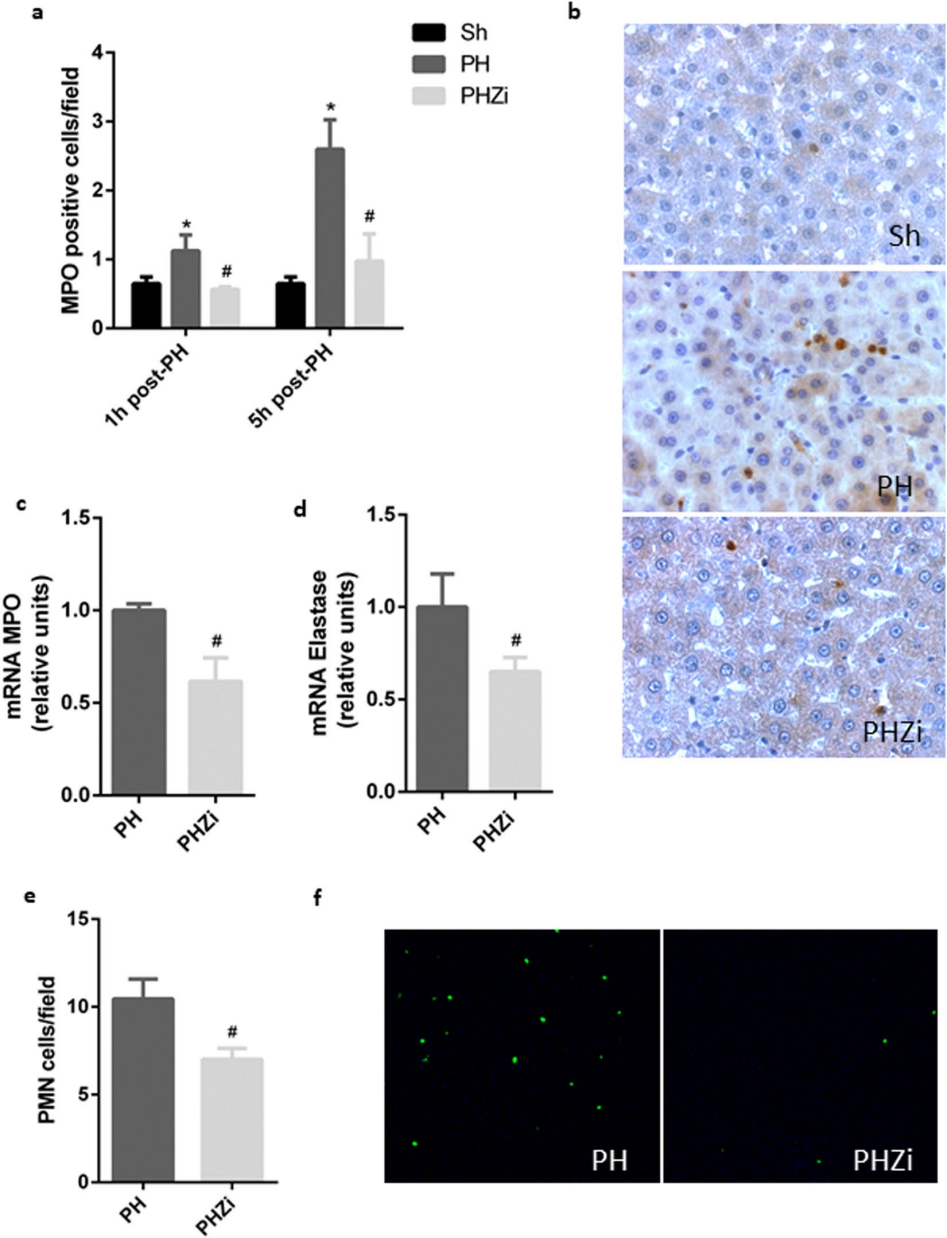
Effect of zileuton treatment on neutrophils recruitment after partial hepatectomy. (**a**) Mieloperoxydase (MPO)-positive cells quantification in liver tissue from Sh, PH and PHZi rats at 1 and 5 h post-PH. (**b**) Representative images of MPO immunostaining 5 h post-PH obtained by optical microscopy (400X). Analysis of mRNA expression of (**c**) MPO and (**d**) elastase in isolated liver leukocytes 5 h after PH. (**d**) Quantification of recruited liver neutrophils 5 h post-PH assessed by immunofluorescence and confocal microscopy. (**e**) Representative images from immunofluorescence detection of neutrophils (100X magnification). Sh: sham animals, PH: partial-hepatectomized animals treated with vehicle, PHZi: PH-animals treated with zileuton 40 mg/Kg body weight. Bars represent mean ± SEM (*n* = 4 per experimental group). *p < 0.05 vs. Sh, ^#^p < 0.05 vs. PH.

It was reported that after PH there is an increase in liver leukocytes, including neutrophils and macrophages. Particularly, neutrophils transiently increase in the liver during the first hours post-PH^31^. As LTB4 is a potent chemoattractant agent for neutrophil granulocytes, we analyzed the impact of reducing hepatic LTB4 levels by zileuton in neutrophils recruitment during regeneration. Analysis of MPO-positive cells showed a significant increase in regenerating livers 1 and 5 h post-PH (Fig. 5a), as previously reported^31^. Zileuton treatment interfered with neutrophils recruitment, as shown by decreased MPO-positive cells in HPZi animals. In accordance, MPO and elastase mRNA expression in isolated liver leukocytes was reduced after zileuton treatment (Fig. 5c,d). Finally, immunofluorescence studies using a specific antibody against rat neutrophils displayed a decrease in neutrophils recruitment in liver tissue from zileuton treated rats (Fig. 5e,f).

### Inhibition of 5-LOX activity by zileuton incremented apoptosis in NPCs after PH

**Figure 6:**
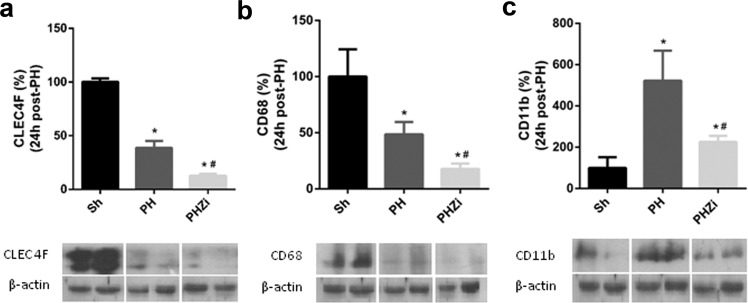
Analysis of macrophages/Kuppfer cells markers in liver NPCs population. Immunoblotting of (**a**) CLEC4F (**b**) CD68 and (**c**) CD11b in NPCs lysates 24 h post-PH. Selected lanes were cropped from different parts of the same gel and they are shown after cropping, aligning and separating them by white space. Full-length blots are available in Supplementary Information. Sh: sham animals, PH: partial-hepatectomized animals treated with vehicle, PHZi: PH-animals treated with zileuton 40 mg/Kg body weight. Bars represent mean ± SEM (*n* = 4 per experimental group). *p < 0.05 vs. Sh, ^#^p < 0.05 vs. PH.

As NPCs are important cytokine and other mediators-producing cells necessary to start and finish liver regeneration, and because Kupffer cells are the only liver cells that possess a metabolically active 5-LOX pathway^7^, we analyzed if 5-LOX inhibition affected these cells. First, we studied CLEC4F, a c-type lectin exclusively expressed by resident Kupffer cells^32^. Figure 6a shows this protein was noticeably reduced in PH animals 24 h post-surgery. At this time, these protein levels were even more reduced in the PH-rats treated with zileuton. Also, CD68 protein levels were reduced 24 h post-PH and even more reduced in the NPCs from animals treated with zileuton (Fig. 6b). By contrast, CD11b levels were augmented in the PH-animals but reduced in the animals treated with zileuton (Fig. 6c). At 1 and 5 h post-PH, CLEC4F, CD68 and CD11b protein levels did not show differences between PH and PHZi NPCs (data not shown).

**Figure 7:**
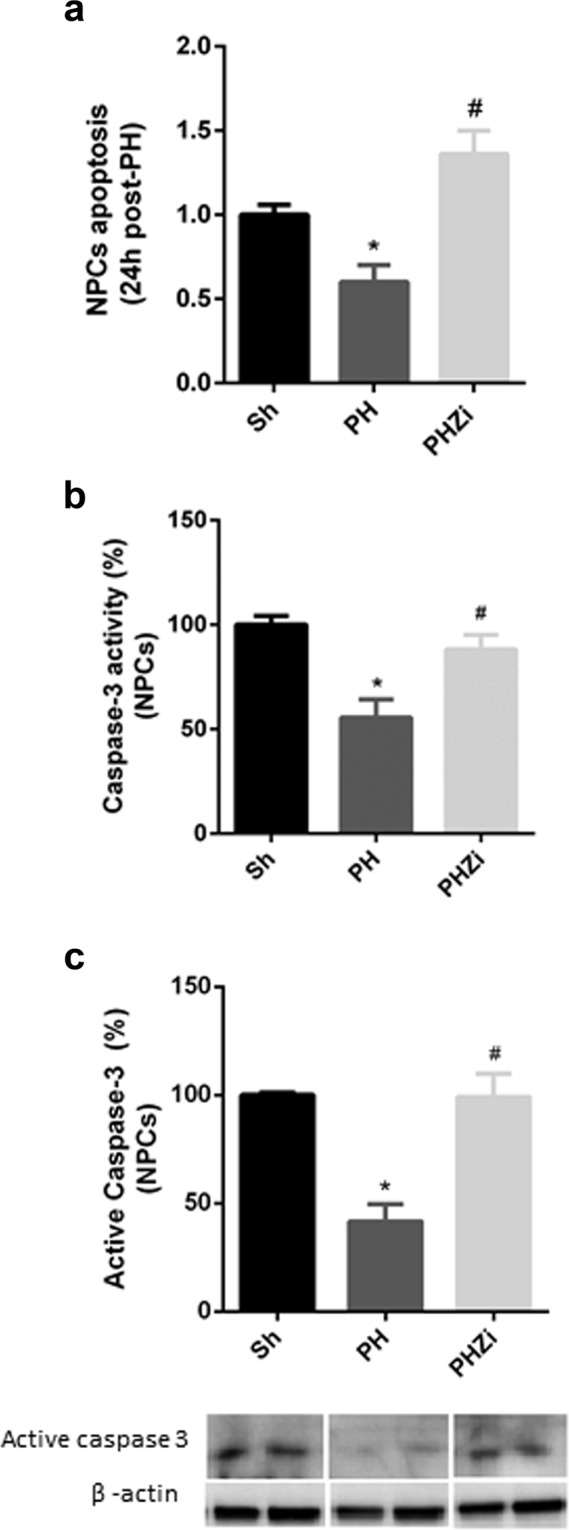
Analysis of NPCs apoptosis 24 h post-PH. Apoptosis was assessed by (**a**) AnnexinV/propidium iodide staining, (**b**) caspase-3 activity, and (**c**) immunoblotting study of active caspase-3. Selected lanes were cropped from different parts of the same gel and they are shown after cropping, aligning and separating them by white space. Full-length blots are available in Supplementary Information. Sh: sham animals, PH: partial-hepatectomized animals treated with vehicle, PHZi: PH-animals treated with zileuton 40 mg/Kg body weight. Bars represent mean ± SEM (*n* = 4 per experimental group). *p < 0.05 vs. Sh, ^#^p < 0.05 vs. PH.

Finally, we studied apoptosis in NPCs 24 h after PH by annexin/propidium iodide staining and also by measuring activity and protein expression of caspase-3. Interestingly, apoptosis of NPCs was reduced in regenerating livers compared to sham operated animals. However, this decrease in apoptosis was not observed in NPCs from zileuton treated PH-rats, showing a significant increment in apoptosis compared with vehicle treated PH-rats (Fig. 7a–c).

## Discussion

The liver has the unmatched capacity for growing back after tissue loss by toxic insults, viral infections or trauma (compensatory hyperplasia). This feature, known as liver regeneration, allows split-liver transplantation, living-donor transplantation and segmental resections for hepatic and metastatic tumors. A better understanding of the many players that cooperate to initiate, sustain and complete liver regeneration is necessary for the comprehension of clinical situations in which liver regeneration is deficient or failed. In this context, the last decades brought a great progress in understanding the kinetic and molecular machinery involved in successful liver regeneration after loss of hepatic mass by two-thirds PH in rodents^2,30^.

The present study provides, for the first time, clear evidences that 5-LOX and LT, particularly LTB4, participate in the regenerative phenomenon after PH in rodents. Previous work of Urade *et al*.^33^ analyzed the effect of 5-LOX inhibition in liver regeneration after PH and, contrary to our results, the authors reported that inhibition of 5-LOX improved liver regeneration. However, in that work authors combined PH with obstructive jaundice and treatment with 5-LOX inhibitor improved liver regeneration because it reduced the inflammatory response induced by bile duct ligation. In fact, they attributed the improvement in liver regeneration to the reduction in LTC4 levels which contribute to hepatic injury during bile flow obstruction. Furthermore, authors administered 5-LOX inhibitor after surgery, so it would not necessarily affect the increase of hepatic LTB4 levels during the first hours post-PH. In other experimental models of hepatic chemical injury with a significant necroinflammatory damage, 5-LOX inhibition or deletion was also beneficial as LT are mediators of inflammation and cell damage^34,35^. On the other hand, in hepatic ischemia/reperfusion injury it was shown that deletion of LTB4 receptor 1 (BLT1) delayed liver repair^36^. Despite many human liver diseases progress in association with innate immunity and necrosis, the study of liver regeneration after PH has the advantage of providing information about the regenerative process relevant only to liver tissue^2,4^. In this context, our study shows a significant increment of hepatic LTB4 levels during the first hours after PH together with an increase in 5-LOX expression. Both increments occurred during the priming phase of liver regeneration and were not observed at later time; still more hepatic LTB4 and 5-LOX expression were significantly reduced at that time. Interestingly, we can consider this new finding as a general event in rodent liver regeneration as it was observed both in rat and mouse liver tissue. Moreover, hepatic LTC4 levels and LTC4-S expression did not show increments at the studied times. On the contrary, they were significantly reduced. Despite we did not explore the mechanisms of LTC4-S regulation, it seems to be reasonable that during liver regeneration hepatic cysteinil LT are maintained at levels that do not cause hepatic injury, given that this kind of eicosanoids are clearly related to tissue damage in liver pathologies such as cholestasis and cirrhosis^37,38^. Additionally, the pattern of 5-LOX protein expression is opposite to that observed and already reported for COX-2 expression during liver regeneration, which is maintained low at early times post-PH but it is augmented at later times^39^. In our study, the profile changes of COX-2 during liver regeneration were not affected by zileuton (data not shown). Regarding this concern, despite it has been previously described that zileuton may affect prostaglandins production under certain circumstances^40^, other publications revealed that zileuton did not change the levels of COX-2 products, even at high doses^41^.

On the other hand, zileuton significantly reduced hepatic LTB4 levels at the moment of hepatectomy and also partially inhibited the increase in hepatic LTB4 content 1 h post-PH. This inhibition produced a delay in the time course of liver proliferation as seen by reduced LW/BW ratio and decreased PCNA and cyclin D1 24 h post-PH, time in which hepatocyte DNA synthesis peaks in rats, unlike mice in which liver regeneration is more retarded (DNA synthesis peaks at 48 h). The delay in liver regeneration in zileuton-treated rats was partially recovered 48 h post-PH. At that time, whereas proliferative index was comparable between groups, LW/BW ratio was not completely recovered.

It has been extensively reported that 5-LOX and its metabolite LTB4 can promote cancer cells proliferation. LTB4 levels are increased in human colon and prostate cancers; also 5-LOX and LTB4 receptor expressions are increased in human pancreatic cancer. Moreover, reduction in LTB4 synthesis by inhibition of LTA4-H reduced esophageal adenocarcinoma development in rats^42^. Particularly in liver cancer, 5-LOX and LTB4 are capable of increasing the transcriptional activity of NF-ĸB through the up-regulation of expression and nuclear translocation of NF-ĸB p65 and the increase of phosphorylation level of ĸBα inhibitor (IĸBα)^16^. In line, our results showed that the diminution in hepatic LTB4 by zileuton is associated with a decrease in the induction of NF-ĸB activity after PH. Moreover, treatment with zileuton maintained IĸBα protein levels to those observed in non-operated animals.

Ohkubo *et al*. showed that LTB4 signaling facilitates liver repair after ischemia/reperfusion injury through activation of epidermal growth factor (EGF) signaling^36^. So, we explored the impact of inhibiting hepatic LTB4 by zileuton on EGF signaling and no differences were observed between PH and PHZi groups (data not shown), indicating that EGF is not involved in zileuton effects.

Considering the established role of LTB4 as an attractant of leukocytes^43^ and the fact that the recruitment of the innate immune cells contributes to liver regeneration^30^, it is possible that the early, transient and controlled increase in hepatic LTB4 after PH favors the recruitment of neutrophils and monocytes/macrophages. So, we studied if reduced hepatic LTB4 levels by zileuton affected the recruitment of these cells. Results showed that zileuton treatment interfered with the recruitment of neutrophils that takes place in the first hours post-PH (Fig. 5) and also interferes with the recruitment of macrophages that takes place at later time (Fig. 6).

Hepatic NPCs encompassing endothelial cells, Kupffer cells/macrophages and stellate cells are critical players in liver regeneration after PH. A recent work showed that when rats were injected with gliotoxin to induce liver NPCs death, mitosis in hepatocytes was absent 24 h after PH^44^. This finding certainly indicates that although hepatocytes themselves are fully capable of repopulating the liver after PH, hepatic NPCs are also required for proper restoration of liver mass. Several lines of evidence demonstrated that Kupffer cells/macrophages activation is beneficial to liver regeneration and provides the initial priming force for hepatocyte proliferation^45^. Liver macrophages have been classified into two subsets: resident CD68^+^ Kupffer cells with phagocytic and bactericidal activities and recruited CD11b^+^ Kupffer cells/macrophages with cytokine-producing capacity. Among these populations, after a PH in mice, CD68^+^ Kupffer cells decreased and CD11b^+^ Kupffer cells/macrophages recruited from the periphery and bone marrow increased^46^. In our study, a reduction in CD68 protein levels and an increment in CD11b together with a decrease in CLEC4F, a specific marker of resident Kupffer cells^32^, were observed 24 h post-PH. Interestingly, treatment with zileuton significantly reduced CD68 and CD11b markers as well as CLEC4F expression in NPCs. Collectively, these results indicate that inhibition of 5-LOX by zileuton affected Kupffer cells/macrophages population and this fact can have a clear impact on the regenerative process. Of all the liver NPCs, Kupffer cells are the only cells that possess a metabolically active 5-LOX pathway and it was demonstrated that inhibition of 5-LOX pathway reduces Kupffer cells growth and induces apoptosis, indicating that a metabolically active 5-LOX pathway is critical for the survival of these cells^7,34^. In line with this, NPCs from zileuton-treated hepatectomized rats showed higher apoptosis than NPCs from control PH–rats. Strikingly, apoptosis of NPCs was significantly reduced 24 h after hepatectomy. This reduction could be important in preserving NPCs for the regenerative process and, as zileuton increased apoptosis levels to those of sham animals, it cannot be discarded that this mechanism is also operating in the delay of regeneration when 5-LOX pathway is inhibited. Additionally, it was reported that NPCs, particularly endothelial cells and macrophages, are the major source of Wnts during liver regeneration^47^. As NPCs were affected by zileuton treatment, this fact can directly impact on the activation of Wnt/β-catenin signaling. Future studies could focus on deepening these findings.

## Conclusions

The present work provides certain evidences that 5-LOX activation and its product LTB4 are involved in the initial signaling events for liver regeneration after PH. In fact, it can be stated that enzymes of the eicosanoids synthesis are differentially expressed during the time-course of liver regeneration. While COX-2 products are crucial at longer times post-PH as was previously established^39^, 5-LOX product, LTB4, is an important signaling mediator for liver regeneration at earlier times. Additionally, we provide evidences that the use of zileuton may affect liver regeneration in situations in which tissue resection is needed. This data is important not only for a better understanding of the mechanisms that take place during liver regeneration but also for considering pharmacological situations in which liver regeneration can be affected. In fact, it was recently published that human hepatocellular carcinoma overexpressed 5-LOX^48^. So, a question emerges regarding if zileuton could be a possible therapeutic strategy to prevent hepatocellular carcinoma recurrence after PH for liver resection. Despite additional studies are necessary to completely delineate the involvement of LTB4, the present results open a new door for the better comprehension of the multiple mechanisms that take place during liver regeneration after PH in which hepatic 5-LOX and LTB4 can be considered new players in the regenerative phenomenon.

## Supplementary information

Supplementary information

## Data Availability

All data generated or analyzed during this study are included in this published article (and in Supplementary Information File).
