# Supplementary material for: Participation of 5-lipoxygenase and LTB4 in liver regeneration after partial hepatectomy
Source: Sci Rep. 2019 Dec 3;9:18176. doi: 10.1038/s41598-019-54652-7 (PMC6890767; doi:10.1038/s41598-019-54652-7)

Participation of 5-lipoxygenase and LTB4 in liver regeneration after partial hepatectomy

Florencia Lorenzetti[1] Marina Cecilia Vera[1] María Paula Ceballos[1] María Teresa Ronco[1] Gerardo Bruno Pisani[2] Juan Alberto Monti[1] Alvaro Lucci[1,2] Carla Gabriela Comanzo[1] Thierry Tordjmann[3] María Cristina Carrillo[1,2] Ariel Darío Quiroga[1,2] María de Luján Alvarez[1,2](*)

[1]Instituto de Fisiología Experimental (IFISE), Facultad de Ciencias Bioquímicas y Farmacéuticas, CONICET, UNR, Suipacha 570 (S2002LRL) Rosario, Argentina

[2]Área Morfología, Facultad de Ciencias Bioquímicas y Farmacéuticas, UNR, Suipacha 570 (S2002LRL) Rosario, Argentina

[3]INSERM U1174, Université Paris Saclay, bât. 443, 91405 Orsay, France

*Corresponding author: María de Luján Alvarez, Ph.D. Instituto de Fisiología Experimental (IFISE), Facultad de Ciencias Bioquímicas y Farmacéuticas, CONICET, UNR. Suipacha 570 (S2002LRL) Rosario, Argentina. E-mail: alvarez@ifise-conicet.gov.ar; Tel: +54 341 4305799

Supplementary materials and methods

Treatment with SC-57461A:

To specifically block LTB4 production, a set of experiments was performed using SC-57461A, a potent and selective inhibitor of LTA4-H with good oral activity in rats and mice [1]. SC-57461A shows a specific inhibition of LTB4 biosynthesis; it does not inhibit 5-LOX, LTC4-S or COX. Male Wistar rats (n = 4) were orally treated with SC-57461A at the dose of 10 mg/Kg body weight, 2 hours before PH. LTA4-H inhibitor was dissolved in 10% DMSO, 90% saline/1% Tween 80. As control, a group of rats (n = 4) received the vehicle of SC-57461A. Hepatic LTB4 content was measured in the removed liver tissue, confirming a significant decrease (- 40%) in hepatic LTB4 levels at the onset of regeneration. Animals were euthanized 24 h post-PH and liver proliferation was assessed by immunohistochemical detection of PCNA.

Mice Samples:

Samples from C57Bl/6 male mice were obtained from Dr. Tordjmann’s Laboratory (INSERM U.757, Universite Paris Sud, Orsay, France). Mice were subjected to partial hepatectomy (PH) which consisted in the removal of two thirds of the liver, including the gallbladder, as described (Mitchell *et al.* 2018). The resected tissue (quiescent liver) was used as control sample (pre-PH). Animals were euthanized at 0.5, 1, 2, 4 and 6 after PH. Liver fragments were stored at -80 °C until use.

Primers sequences:

Rat 5-LOX Forward	5´ ACCTGACGGTGGTGATCTTC 3´
Rat 5-LOX Reverse	5´ GATCCAGGAGCACCAGTCAT 3´
Rat LTA4-H Forward	5´ ATCCCACGATCAAGCTGTCC 3´
Rat LTA4-H Reverse	5´ CAGATCTTTCCCCACCAGCA 3´
Rat LTC4-S Forward	5´ CCTACAGGTGATCTCTGCGC 3´
Rat LTC4-S Reverse	5´ GCAATTTACCTGGGCTCGGA 3´
Rat IL-6 Forward	5' AGTGGCTAAGGACCAAGACC 3'
Rat IL-6 Reverse	5’ TAGCACACTAGGTTTGCCGAG 3'
Rat GAPDH Forward	5´ ATGACTCTACCCACGGCAAG 3´
Rat GAPDH Reverse	5´ GATCTCGCTCCTGGAAGATG 3´
Rat MPO Forward	5´CAAAGCCTACAAGGAGCGGA 3´
Rat MPO Reverse	5´ACCGGCTGCTTGAAATACGA 3´
Rat Elastase Forward	5´CAGCGCATCTTTGAGAACGG 3´
Rat Elastase Reverse	5´TGGTAGCTGAGCCATTGAGC 3´
Rat Wnt2 Forward	5´TGTGACAATGTGCCAGGTCT 3´
Rat Wnt2 Reverse	5´CAATGGCACGCATCACGTC 3´
Rat Wnt9b Forward	5´ACACCAGCTATTCTGGCACC 3´
Rat Wnt9b Reverse	5´GGTGCTCAGACAGGAAGCAT 3´
Mouse 5-LOX Forward	5´ CACTGTTCTCTTTCGATCAACAAT 3´
Mouse 5-LOX Reverse	5´ GGGGCAAAGACCTTGTCA 3’
Mouse LTA4-H Forward	5´ AGATGGTTACGGCTCTGCAT 3´
Mouse LTA4-H Reverse	5´GGCAGCGAGATCCTTGAATAGA 3´
Mouse LTC4-S Forward	5´CTCTTCTGGCTACCGTCACC 3´
Mouse LTC4-S Reverse	5´AAGCCCTTCGTGCAGAGAT 3´

Supplementary figures

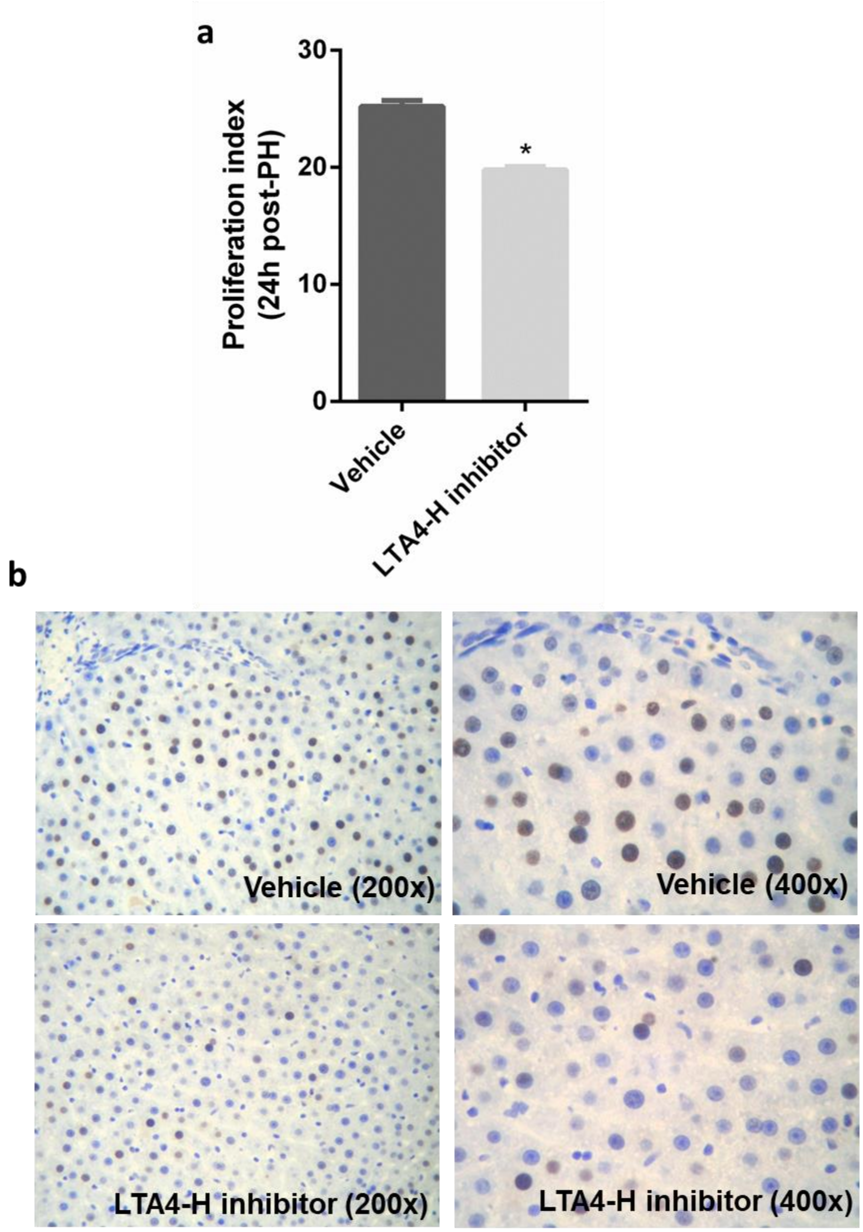

Supplementary Fig 1: Effect of LTA4-H inhibition in rat liver proliferation 24 h post-PH. (a) Proliferative index determined as PCNA-positive cells per 100 hepatocytes counted in 10 high-power fields. (b) Representative images of PCNA immunohistochemistry from liver tissue obtained by optical microscopy. Bars represent mean ± SEM (*n* = 3). *p<0.05

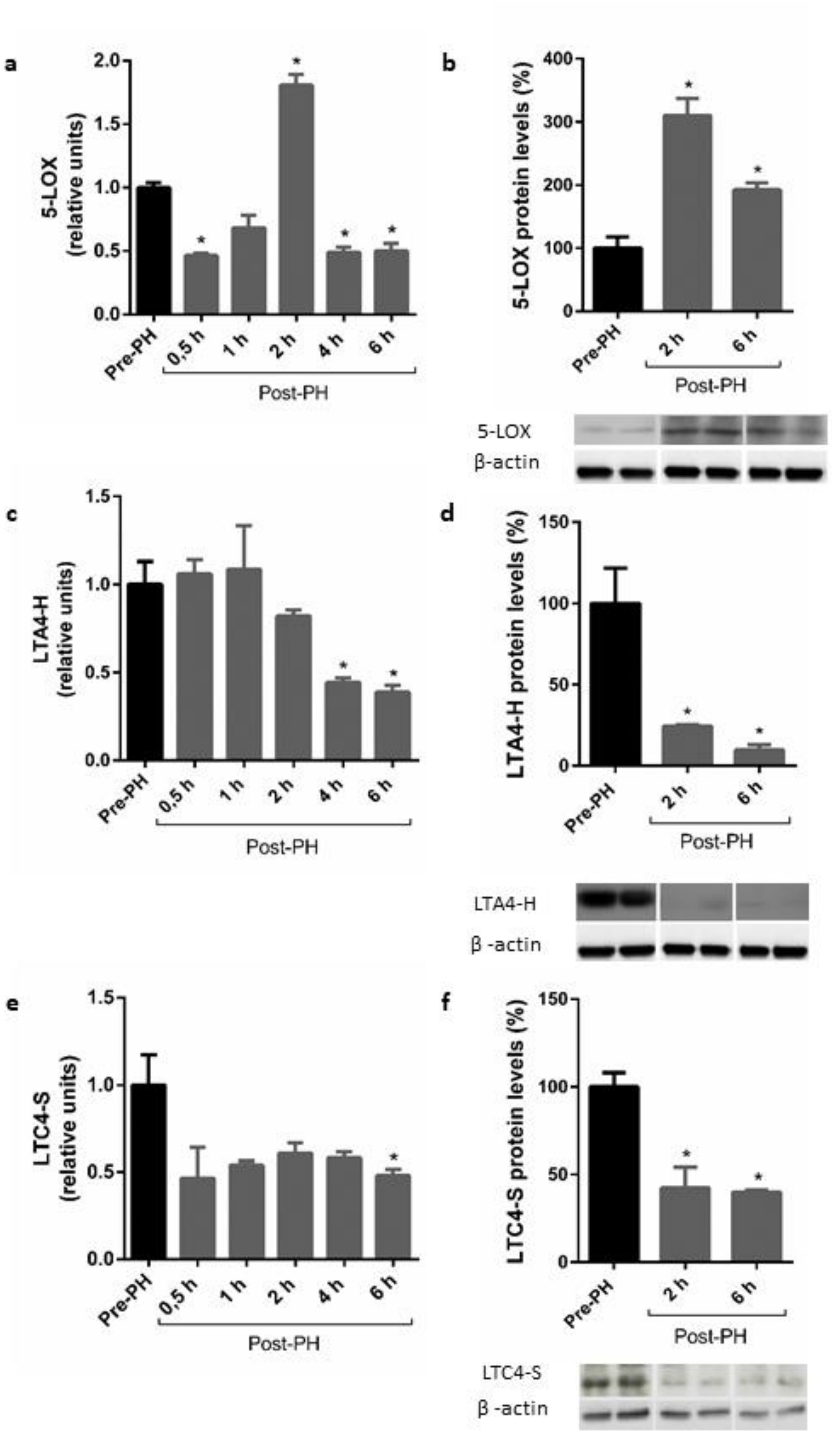

Supplementary Fig. 2: mRNA and protein expression of main enzymes involved in leukotrienes synthesis measured in C57Bl/6 male mice liver samples. RT-qPCR analysis of

(a) 5-LOX, (c) LTA4-H and (e) LTC4-S. Immunoblotting study of (b) 5-LOX, (d) LTA4-H and (f) LTC4-S. Selected lanes were cropped from different parts of the same gel and they are shown after cropping, aligning and separating them by white space. Full-length blots are available in the raw data section. Pre-PH: Removed tissue (at time zero of surgery), Post-PH: partial hepatectomized animals. Bars represent mean ± SEM (*n* = 5). *p<0.05 vs. Pre-PH

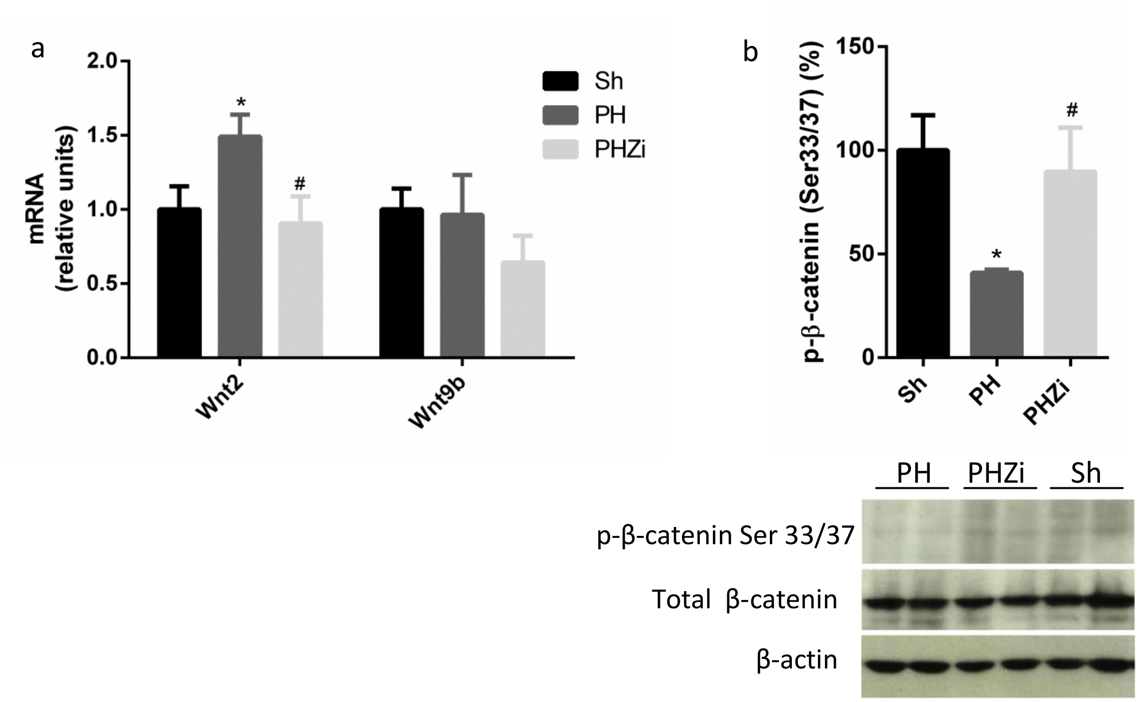

Supplementary Fig. 3: Alteration of Wnt/β-catenin pathway during liver regeneration after inhibition of 5-LOX by zileuton. (a) RT-qPCR analysis of the main Wnts involved in liver regeneration (Wnt2 and Wnt9b) in NPCs 1 h post-PH. (b) Immunoblotting of inactive (phosphorylated) p-β-catenin (sc-16743-R, Santa Cruz) and total β-catenin (610154, BD Biosciences) in total lysates 24h post-PH. Sh: Sham animals, PH: partial-hepatectomized animals treated with vehicle, PHZi: PH-animals treated with zileuton 40 mg/Kg body weight. Bars represent mean ± SEM (*n* = 4). *p<0.05 vs. Sh, #p<0.05 vs. PH

Supplementary Bibliography

1. Kachur, J. F. *et al.* Pharmacological characterization of SC-57461A (3-[methyl[3-[4-(phenylmethyl)phenoxy]propyl]amino]propanoic acid HCl), a potent and selective inhibitor of leukotriene A(4) hydrolase II: in vivo studies. *J. Pharmacol. Exp. Ther.* **300**, 583–7 (2002).

Raw data:

Raw data Fig.1f:

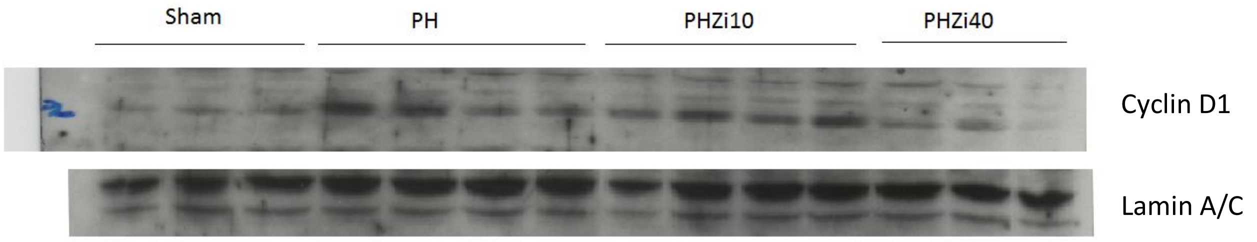

Raw data Fig. 3a:

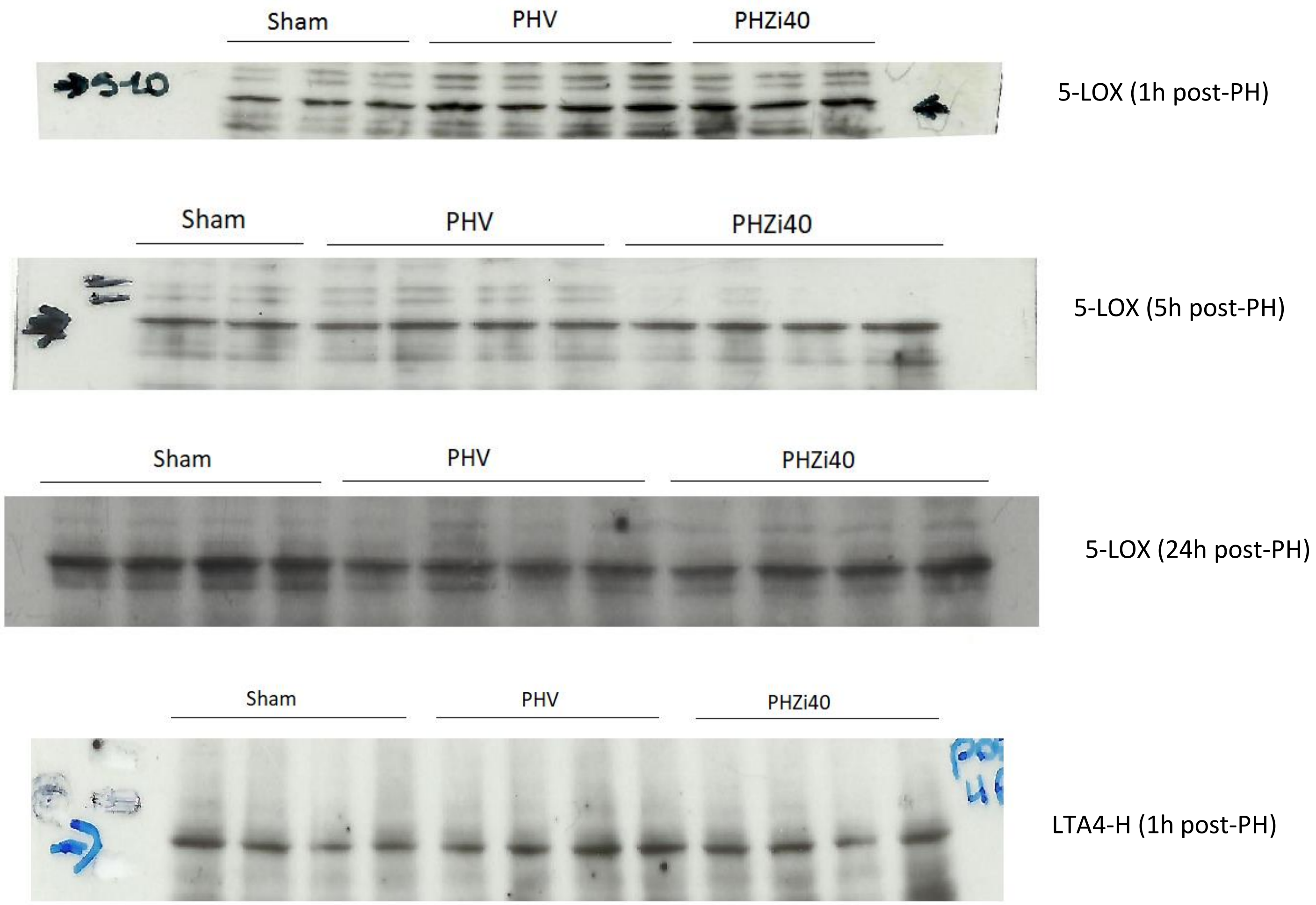

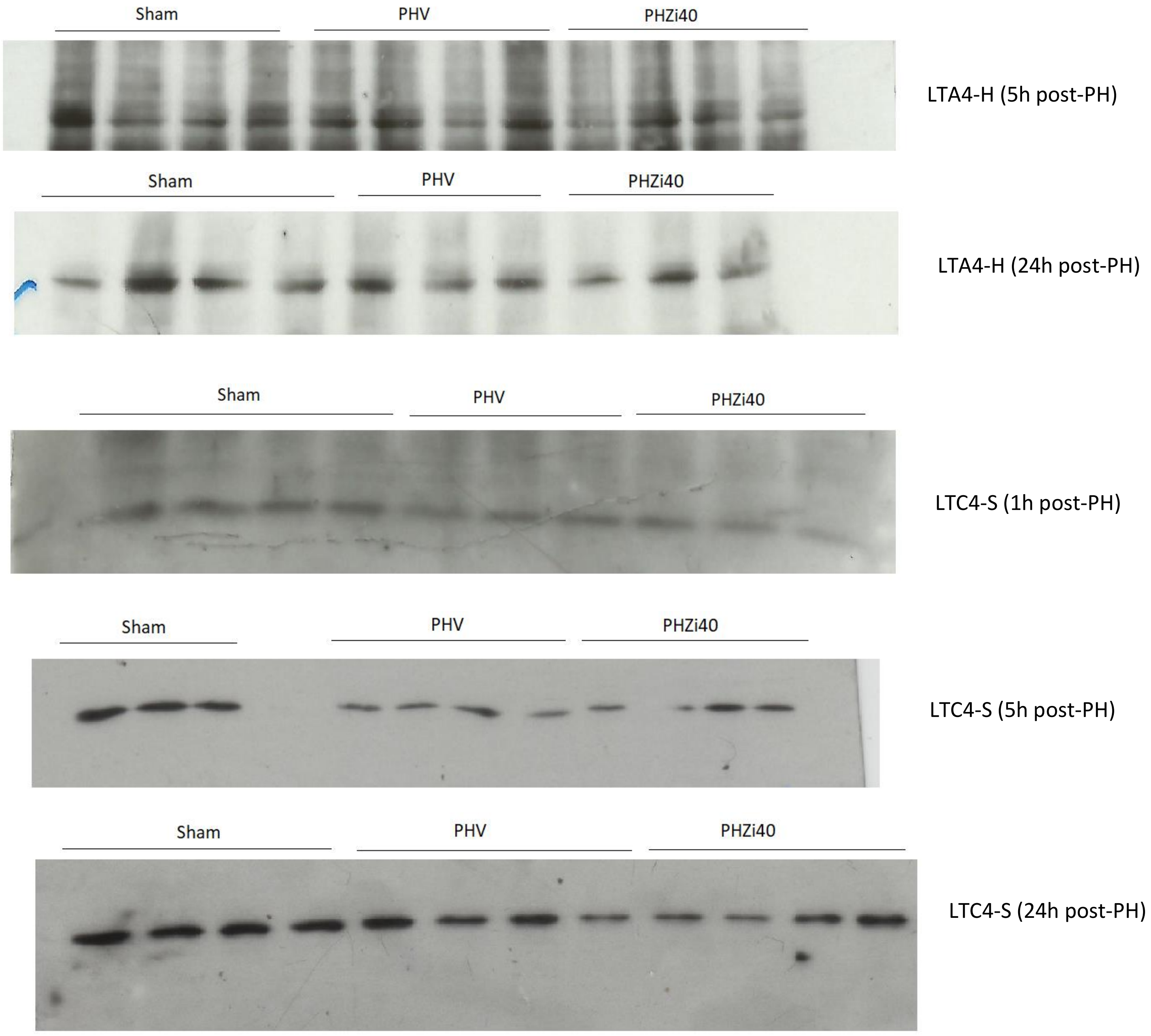

Sham
PHV
PHZi40
LTA4-H (5h post-PH)
Sham
PHV
PHZi40
LTA4-H (24h post-PH)
Sham
PHV
PHZi40
LTC4-S (1h post-PH)
Sham
PHV
PHZi40
LTC4-S (5h post-PH)
Sham
PHV
PHZi40
LTC4-S (24h post-PH)

Raw data Fig. 4b:

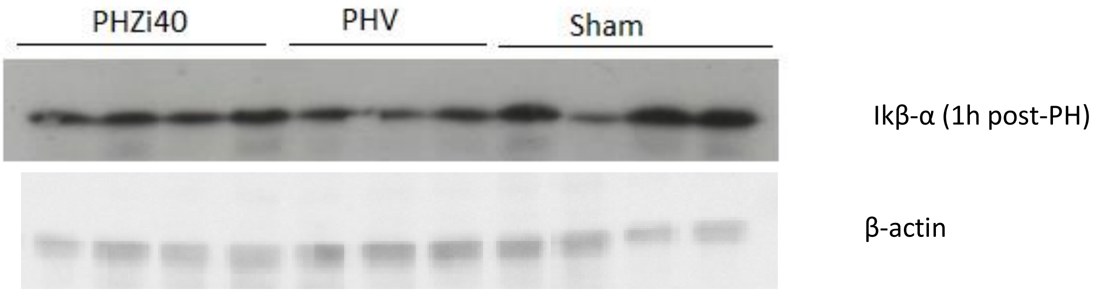

Raw data Fig. 5a:

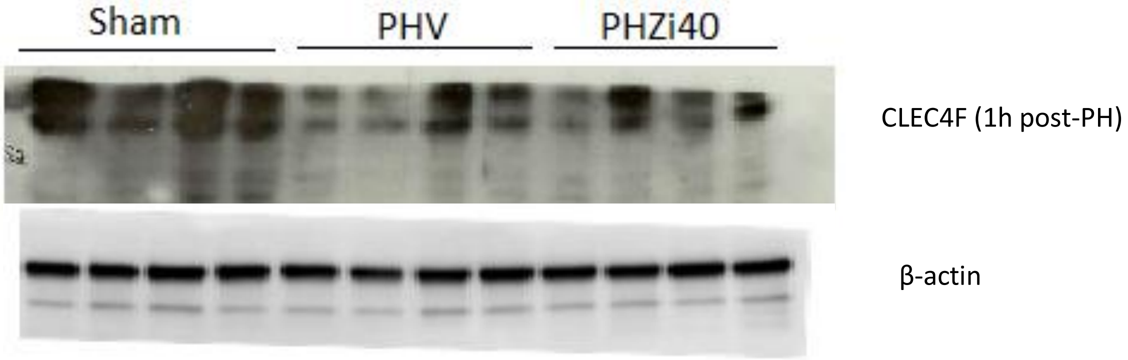

Raw data Fig. 5b:

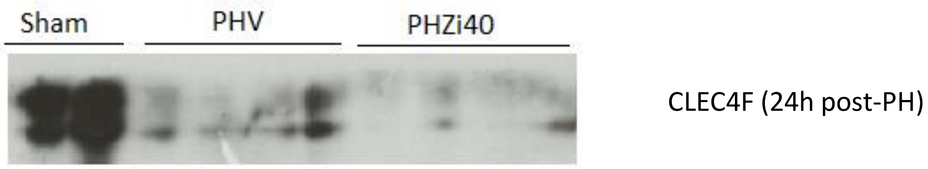

Raw data Fig. 5c:

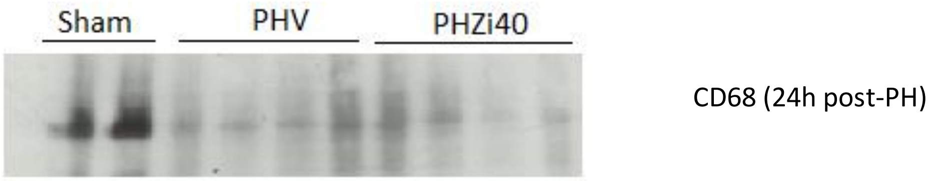

Raw data Fig 5d:

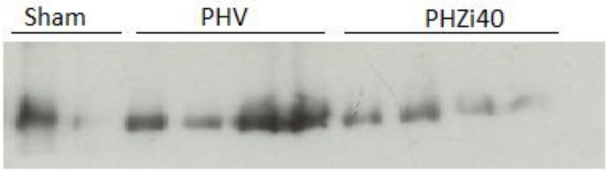

CD11b (24h post-PH)

β-actin Fig 5b, c, and d:

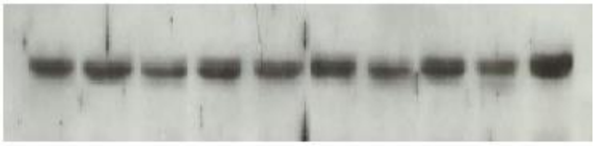

β-actin

Raw data Fig. 6c:

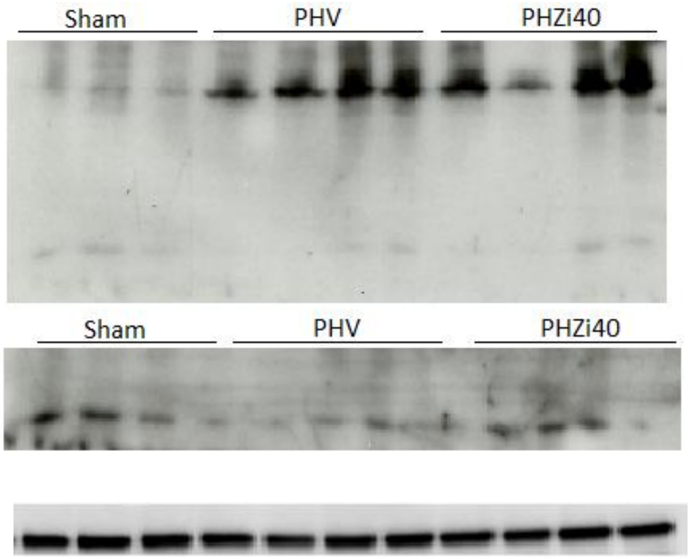

Procaspase-3 (24h post-PH)

Active caspase-3 (24h post-PH)

Active caspase-3 (24h post-PH)

(more exposed)

β-actin

Raw data Supplementary Figure:

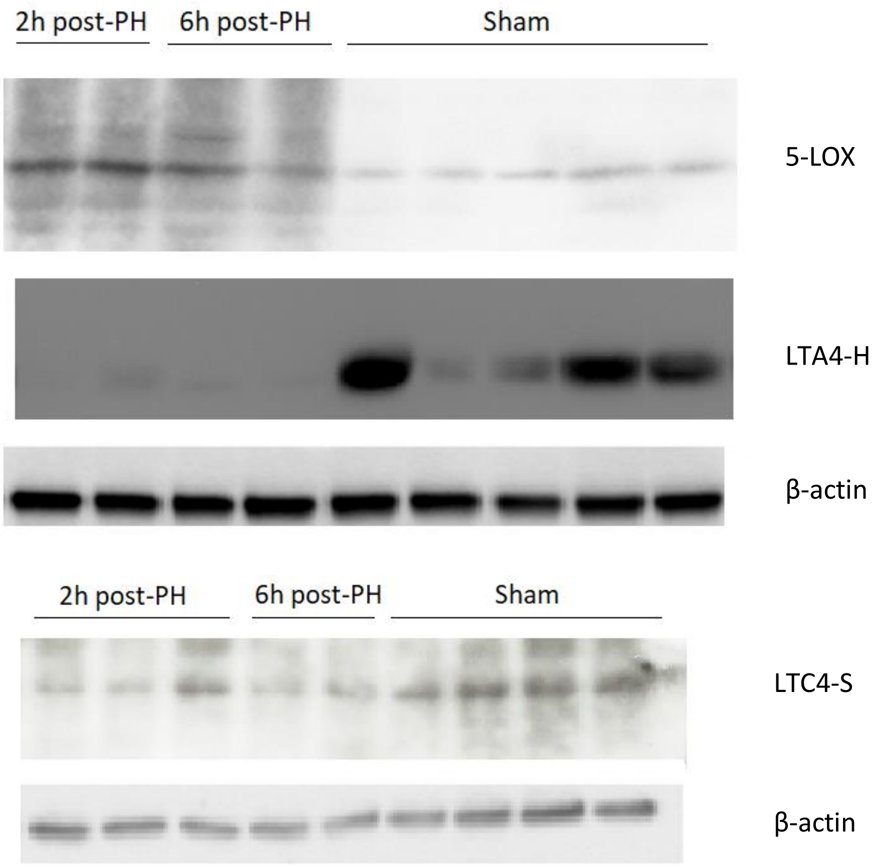

Supplement: Supplementary file 1 — Supplementary information [file 41598_2019_54652_MOESM1_ESM.pdf]
